# Robust ZIF-67/AC/PDMS hybrid nanocomposite for superhydrophobic steel protection

**DOI:** 10.1038/s41598-026-49485-0

**Published:** 2026-05-09

**Authors:** Huda F. Khalil, Mohamed Abdel Rafea, Mervette El-Batouti, Sara Gad, Mohamed H. Eisa, Sherif G. Elsharkawy

**Affiliations:** 1 Electronic Materials Department, Advanced Technology and New Material Institute (ATNMI), City of Scientific Research and Technological Applications (SRTA-City), Alexandria, 21934 Egypt; 2 Department of Physics, College of Science, Imam Mohammad Ibn Saud Islamic University (IMSIU), Riyadh, 11623 Saudi Arabia; 3 Chemistry Department, Faculty of Science, Alexandria University, Alexandria, 21934 Egypt; 4 Basic and Applied Sciences, College of Engineering and Technology, AASTMT, 21934 Alexandria, Egypt

**Keywords:** Anticorrosion, Cassie-Baxter state, Contact angle, Hybrid nanocomposites, Surface roughness, Chemistry, Materials science

## Abstract

A superhydrophobic coating synthesized from Cobalt-based Zeolitic Imidazolate Framework (ZIF-67), Activated Carbon (AC), and Polydimethylsiloxane (PDMS) was successfully fabricated on a steel substrate. This coating was synthesized with concentration ratios S2 (1 wt% ZIF-67), S3 (3 wt% ZIF-67), S4 (5 wt% ZIF-67) and S5 (7 wt% ZIF-67), respectively. XRD and HR-TEM were applied to examine the crystalline structure and morphology of the synthesized nanocomposite. The results revealed S4 (5 wt% ZIF-67) composite as an optimized coating as it performed an exceptional surface wettability with a water contact angle (WCA) of 170° and superior chemical stability. Long-time laps of immersion tests in different pH range (3–11) nominated the S4 coating for retaining superhydrophobic integrity over 48 h, even placed in aggressive acidic or alkaline media. Electrochemical Impedance Spectroscopy (EIS) confirmed a robust corrosion protection performance of the S4 coating, as it recorded a high charge transfer resistance (R_ct_) reaching 5 × 10^5^ Ω⋅cm^2^. This enhanced performance is attributed to the modified roughness resulting from the ZIF-67/AC framework and the low surface energy of the PDMS matrix that stabilized a persistent Cassie-Baxter state. These results demonstrate the ZIF-67/AC/PDMS nanocomposite as a promising strategy for sustained corrosion protection of steel in severe industrial environments.

## Introduction

Steel structure is one of the most widely used materials in modern industry. This is mainly due to its excellent mechanical properties as well as its cost effectiveness. However, its high susceptibility to corrosion in diverse environments remains a critical challenge for all fields of applications leading to significant economic losses and safety concerns. Superhydrophobic (SHP) coatings^1–3^ with water contact angles greater than 150° has taken great attention as they build an effective strategy for corrosion mitigation. These coatings perform to trap air layer generating air pockets beneath which is defined as ‘Cassie-Baxter’ state^4^. Transition between different wetting states governing the anti-corrosive performance of SHCs. According to the Cassie-Baxter model, the entrapment of air within the micro-nanoscale hierarchical roughness creates a plastron (an air cushion) at the interface between the coating and the electrolyte. This air layer acts as a physical barrier that prevents corrosive species, such as $\:{Cl}^{-}$ and O_2_, from reaching the metallic substrate^5^. Mathematically, the apparent contact angle θ on such surfaces is described by the Eq.^6^:

1 $$\:\mathrm{cos}\theta\:={f}_{s}\:\left(\mathrm{cos}\theta\:+1\right)-1$$

Recent advancements in materials field have introduced Zeolitic Imidazolate Framework, (ZIF-67)^7,8^, as promising modified surfaces candidates due to their high porosity, tunable structure, and exceptional chemical stability^9^. In recent years, several studies have explored the integration of Metal-Organic Frameworks (MOFs) into polymeric matrices to enhance barrier properties; for instance, ZIF-8 and ZIF-67 have demonstrated significant potential in improving the tortuosity of the diffusion path for corrosive ions^10^. Furthermore, carbonaceous materials like Activated Carbon (AC) have been utilized to reinforce the mechanical integrity and surface roughness of composite coatings^11^. However, a significant gap remains in the literature regarding the synergistic combination of ZIF-67 and AC to create a multi-level hierarchical structure that can withstand prolonged immersion in aggressive saline media. Integrating MOFs with carbonaceous materials like Activated Carbon (AC) can further enhance the surface roughness and mechanical integrity of the coating. However, achieving a balance between superhydrophobicity and sustained corrosion protection in aggressive pH environments remains a hurdle for many MOF-based coatings^12^. Recent research on Metal-Organic Frameworks (MOFs), particularly Zeolitic Imidazolate Framework, (ZIF-67) and its derivatives for the adsorption of antibiotics^13,14^. Zeolitic imidazolate framework-8 (ZIF-8) layers followed by Polydimethylsiloxane (PDMS) were employed through modification fabrication method for constructing robust, wearable, and fluorine-free superhydrophobic surfaces^15^. Great focus is being directed nowadays for researches adopting superhydrophobic cotton fabrics for oil water separation techniques^16^. The novelty of this work lies in the strategic design of a ZIF-67/AC/PDMS hybrid architecture to overcome the limitations of single-filler systems. Unlike previous studies that often require complex multi-step fabrication, our approach utilizes a facile dip-coating technique to leverage the MOF/Carbon hybrid synergy. In this system, ZIF-67 a subclass of metal-organic frameworks (MOFs) with a unique dodecahedral crystalline structure provides the necessary hierarchical roughness, moving beyond its conventional applications in adsorption and catalysis. Simultaneously, Activated Carbon (AC) acts as a reinforcing filler to create a tortuous barrier, while PDMS ensures low surface energy and structural integrity. This dual-action mechanism provides physical air trapping (plastron stability) and enhanced chemical barrier resistance for superior steel protection. In this work, we systematically investigate the effect of ZIF-67 concentration on the coating’s morphology, wettability, and electrochemical performance, providing a facile approach to designing multifunctional coatings for advanced corrosion protection.

## Materials and experimental procedures

### Materials

Cobalt (II) nitrate hexahydrate (Co(NO_3_)_2_.6H_2_O, 98% purity) and 2-Methylimidazole (MeIm, 99% purity) were purchased from Sigma-Aldrich (USA). Activated Carbon (AC) (DARCO, 100 mesh) and n-hexane (reagent grade, ≥ 95%) were obtained from Sigma Aldrich (Germany). The PDMS (Sylgard 184) and its curing agent were supplied by Dow Corning (USA). Anhydrous methanol (99.8%) was used as the primary solvent for MOF synthesis (Table 1).

**Table 1:** Materials chemical formula for samples (1, 3, 5 and 7wt.%) ZIF67/AC/PDMS hybrid (S2, S3, S4 and S5).

Material name	Chemical formula	Manufacturer	Weight/quantity	Purpose
Cobalt(II) nitrate hexahydrate	Co(NO_3_​)_2_​⋅6H_2_​O (98%)	Sigma-Aldrich (USA)	Variable (ZIF Synthesis)	MOF Metal Precursor
2-Methylimidazole	C_4_​H_6_​N_2_​ (99%)	Sigma-Aldrich (USA)	Variable (ZIF Synthesis)	MOF Organic Ligand
Activated Carbon (AC)	Powdered (DARCO)	Sigma-Aldrich (Germany)	1.0–5.0 wt%	Surface Roughness Filler
Polydimethylsiloxane (PDMS)	Sylgard-184 Silicone Elastomer	Dow Corning/Sigma	10:1 Base-to-Curing Agent	Hydrophobic Binder
n-Hexane	Reagent Grade (≥ 95%)	Sigma-Aldrich (USA)	As needed	Solvent for Dilution
Methanol	Anhydrous (99.8%)	Sigma-Aldrich (USA)	As needed	Synthesis Medium

### Substrate pre-treatment

First, the surfaces of the steel substrates ($\:20\:\mathrm{m}\mathrm{m}\:\times\:\:20\:\mathrm{m}\mathrm{m}\:\times\:\:2\:\mathrm{m}\mathrm{m}$) were polished using a series of silicon carbide emery papers (from 400 to 2000 grit) to achieve a uniform surface finish. Then, the substrates underwent a multi-stage ultrasonic cleaning process using ethanol and deionized water for 20 min each. This preparation phase was crucial to remove any residual oils or oxidation layers that might impede the subsequent bonding of the PDMS matrix.

### Synthesis of ZIF-67/AC hybrid filler

The ZIF-67 nanocrystals were synthesized through a room-temperature chemical coordination method. Initially, 0.45 g of Cobalt (II) nitrate hexahydrate and 1.26 g of 2-Methylimidazole were dissolved in separate methanol solutions. To integrate the carbonaceous framework, a calculated amount of Activated Carbon (AC) was added to the cobalt precursor solution under high speed stirring^17^. Once the solutions were combined, the reaction was allowed to proceed for 24 h at room temperature. The resulting hybrid precipitate was then collected via centrifugation at 8000 rpm, washed three times with ethanol to remove unreacted species, and dried at 60 °C for 12 h to yield the final ZIF-67/AC filler. For the functional coatings fabrication (S2–S5), specific weight percentages of the ZIF-67/AC hybrid (1, 3, 5, and 7 wt%) were dispersed into a diluted Polydimethylsiloxane (PDMS) solution (10 wt% in n-hexane) based on the total mass of the PDMS polymer matrix (base + curing agent). Then sonicated for 45 min to break any nanoparticle agglomerates. The pre-treated steel substrates were vertically immersed into the resulting stable suspension for 2 min and then withdrawn at a constant speed of 50 mm/min to ensure a uniform film thickness via the drainage-evaporation mechanism. Finally, the samples were thermally cured at 80 °C for 2 h to promote the polymerization of PDMS and secure anchoring of the fillers. The average coating thickness was measured to be approximately [$\:35\pm\:5\:\mu\:m$] using cross-sectional SEM analysis. Detailed compositions and the specific mass of AC in each coating are summarized in Table 2.

**Table 2:** Composition details and AC mass for the fabricated coatings.

Sample ID	Hybrid Filler (wt%)	Mass of ZIF-67/AC (g)	Mass of AC (mg)	Coating Thickness (µm)
S2	1%	0.1	10	[30]
S3	3%	0.3	30	[33]
S4	5%	0.5	50	[36]
S5	7%	0.7	70	[40]

### Characterization techniques

The crystalline structure and phase purity were analyzed using X-ray diffraction (XRD, Bruker D8 Advance, Germany) with Cu-K$\:\alpha\:$ radiation ($\:\lambda\:$= 1.5406 Å) operated at 40 kV and 40 mA. The surface morphology and elemental composition were investigated via Field Emission Scanning Electron Microscopy (FESEM, JEOL JSM-7600 F, Japan) equipped with Energy Dispersive X-ray Spectroscopy (EDX). While the morphological features and lattice fringes were investigated via high Resolution Transmission Electron Microscopy (HR-TEM, model JEOL JEM-2100 F, Tokyo, Japan) operating at an accelerating voltage of 200 kV. The chemical functional groups were identified using Fourier Transform Infrared Spectroscopy (FTIR, Shimadzu, Japan) in the range of 400–4000 cm^-1^. Water contact angles (WCA) were measured using a contact angle goniometer with a 5 µL water droplet. Electrochemical corrosion tests were conducted using a BioLogic SP-150 potentiostat (France) in a 3.5 *wt*% NaCl solution at ambient temperature ($\:25\pm\:2^\circ\:c$ ). A standard three electrode cell was utilized, consisting of the coated steel as the working electrode, a Platinum (Pt) wire as the counter electrode, and a Saturated Calomel Electrode (SCE) as the reference electrode. Before each measurement, the samples were immersed in the electrolyte for 1 h to reach a stable Open Circuit Potential (OCP). PDP curves were recorded at a scan rate of 1 mV/s within a potential range of $\:\pm\:250\:mV$ vs. Eocp.

## Result and discussion

### X-ray diffraction (XRD) analysis

**Fig. 1:**
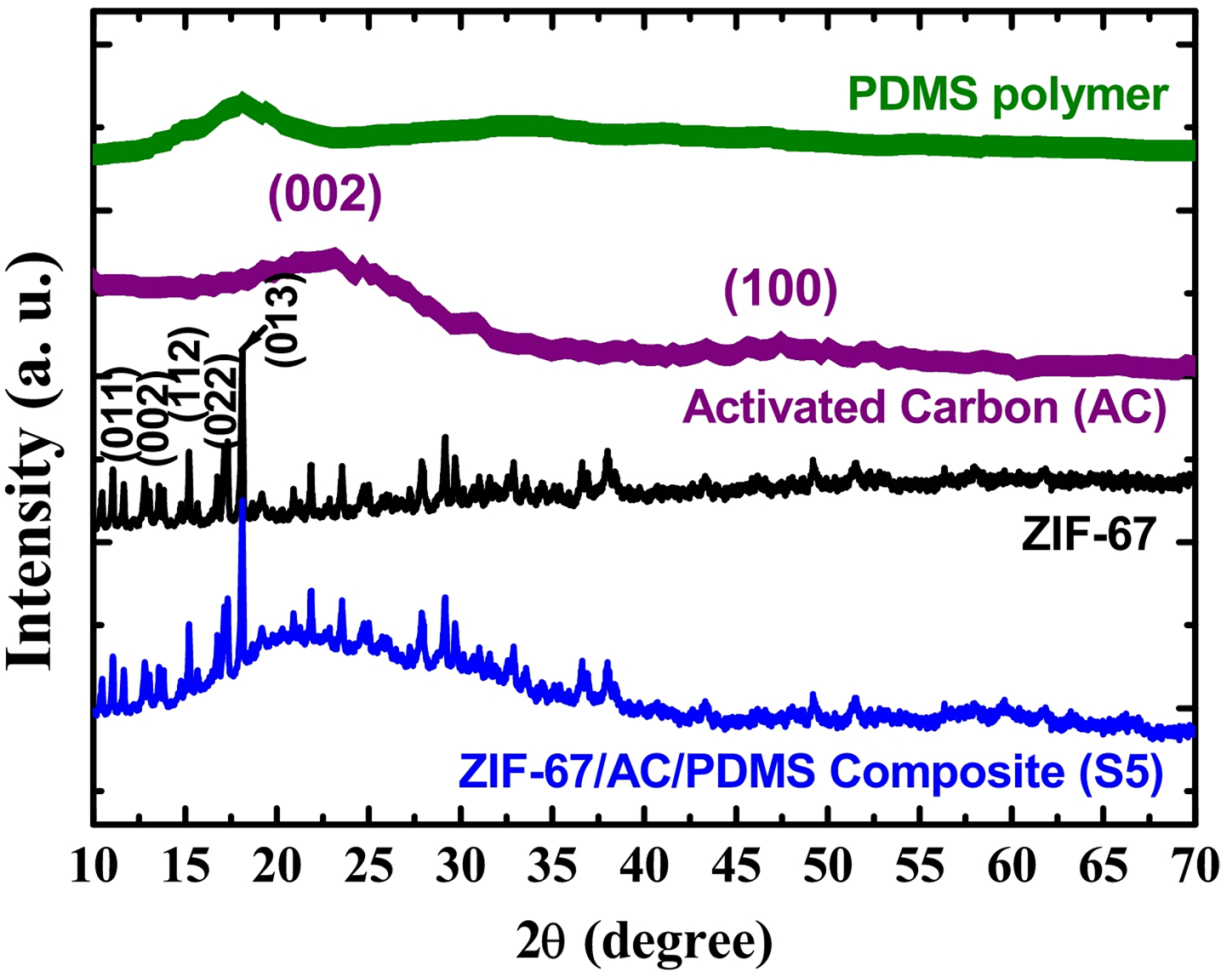
XRD patterns of pristine PDMS polymer, Activated Carbon (AC), ZIF-67 nanocrystals, and the optimized ZIF-67/AC/PDMS composite (S5).

The crystalline structure and phase purity of the materials were characterized using X-ray diffraction (XRD), as shown in Fig. 1. The XRD pattern of the pure ZIF-67 (black curve) exhibits sharp and intense diffraction peaks at $\:2\theta\:$ values of 10.4^o^, 12.7^o^, 14.7^o^, 16.5$\:^\circ\:\:$and 18.1°, which are indexed to the (011), (002), (112), (022), and (013) planes, respectively. These peaks represent the characteristic fingerprints^18^ of the sodalite type zeolitic imidazolate framework (matching JCPDS card no. 00-062-1292). These results confirm the successful synthesis of highly crystalline ZIF-67 nanocrystals. The XRD spectrum of the Activated Carbon (AC) (purple curve) displays a broad diffraction peak at approximately $\:2\theta\:=\:24.5^\circ\:,$ corresponding to the (002) plane of the disordered carbon framework. Another weak and broad peak appears around$\:\:2\theta\:=\:44^\circ\:$, indexed to the (100) plane, further confirming the amorphous and porous nature of the AC carrier^19^. For the PDMS polymer (green curve), a characteristic broad amorphous halo is observed at$\:\:2\theta\:\:=\:12^\circ\:$, indicating the typical amorphous state of the polydimethylsiloxane chains.

As shown in the XRD pattern of the optimized composite (S5, blue curve), the characteristic diffraction peaks of ZIF-67, particularly the intense peak at $\:2\theta\:$ ≈18.1° (013), remain clearly visible. In the final composite samples, the presence of all constituent peaks, superimposed on the broad amorphous features of the AC/PDMS matrix, specifically the sharp ZIF-67 signals and the broad AC/PDMS features, indicates that the ZIF-67 framework maintained its structural integrity throughout the coating fabrication process^20^. Furthermore, the average crystallite size of ZIF-67 was calculated using the Scherrer equation based on the prominent peak at 18.1°, yielding a value of approximately 28 nm. This result is in high correlation with the average particle size of 30 nm observed in the TEM analysis, confirming the high crystallinity and phase purity of the hybrid system. No additional impurity peaks were detected, confirming the successful integration of the ZIF-67/AC/PDMS hybrid system.

### Morphological and particle size analysis

**Fig. 2:**
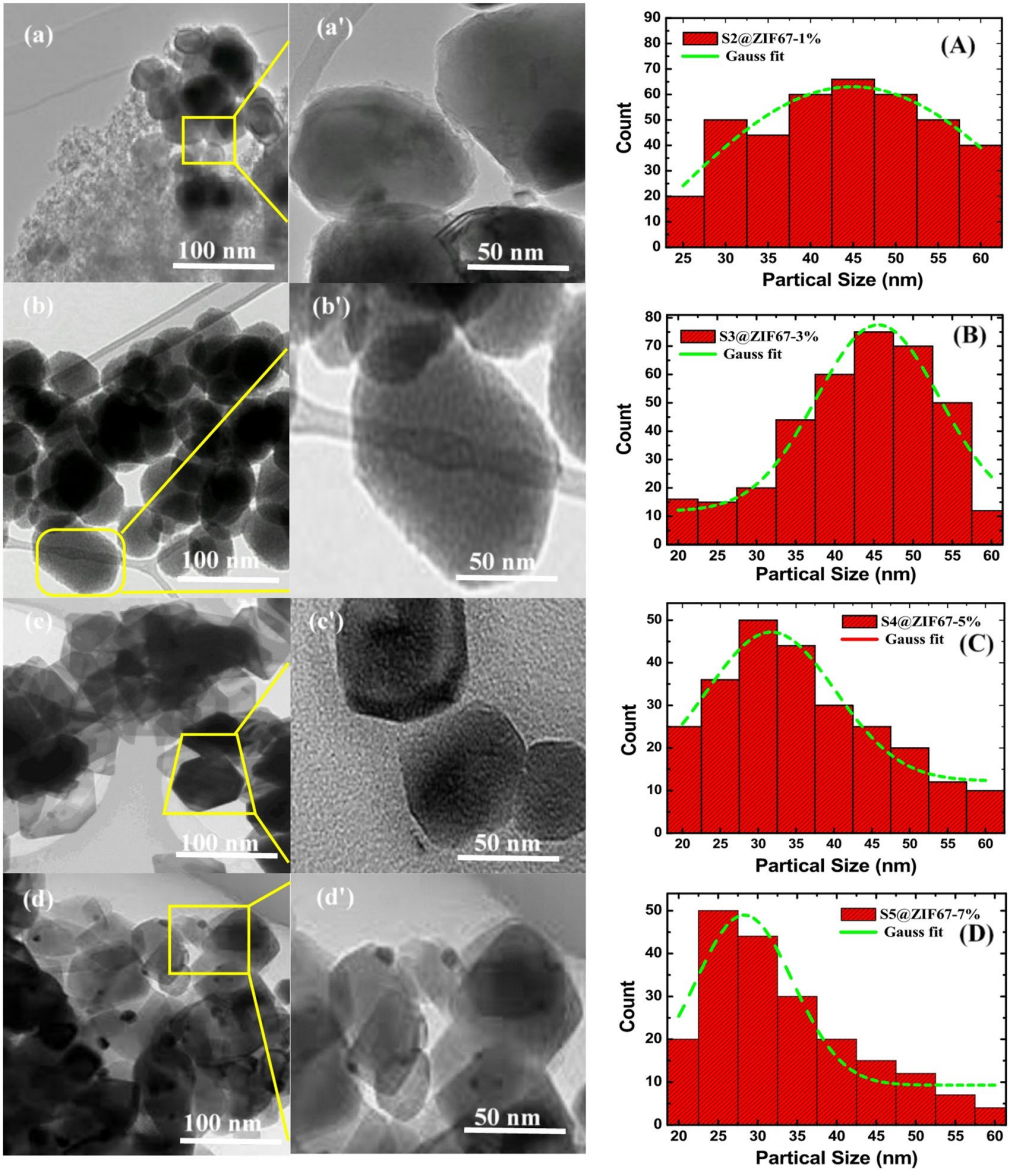
TEM for all samples S2, S3, S4 and S5 at magnification (**a**–**d**) 100 nm align, (**a'**–**d'**) 50 nm align, and (**A**–**D**) particle size analysis.

The surface morphology and internal structure of the synthesized ZIF-67/AC/PDMS composite were investigated using Transmission Electron Microscopy (TEM). As illustrated in the high resolution nanographs. Figure 2 (a, b, c and d) at magnification 100 nm align show that the ZIF-67 nanocrystals exhibited a well-defined, sharp edged rhombic morphology^21–23^. A thorough inspection at 50 nm align level, Fig. 2 (a’, b’, c’, d’) confirmed the ZIF-67 polyhedral geometry high-quality crystalline nanostructure of the composite. These images reveal that these nanocrystals are effectively coated onto the Activated Carbon (AC) sheets, creating a hierarchical nanostructure. Particle Size Analysis (PSA) was conducted for all samples illustrated in Fig. 2 (A, B, C and D). The resulting histograms show narrow size distributions with distinct peaks within range 27–46 nm reflecting excellent agreement with the TEM observations. To further investigate the crystallinity, high-resolution TEM (HR-TEM) was conducted. The images revealed clear lattice fringes with an interplanar spacing of 0.25 nm, corresponding to the (112) crystallographic plane of ZIF-67. Additionally, the SAED pattern exhibited sharp diffraction rings, confirming the highly crystalline and polycrystalline nature of the MOF particles embedded within the AC/PDMS framework. These findings correlate perfectly with the XRD data, ensuring the structural stability of the filler.

**Fig. 3:**
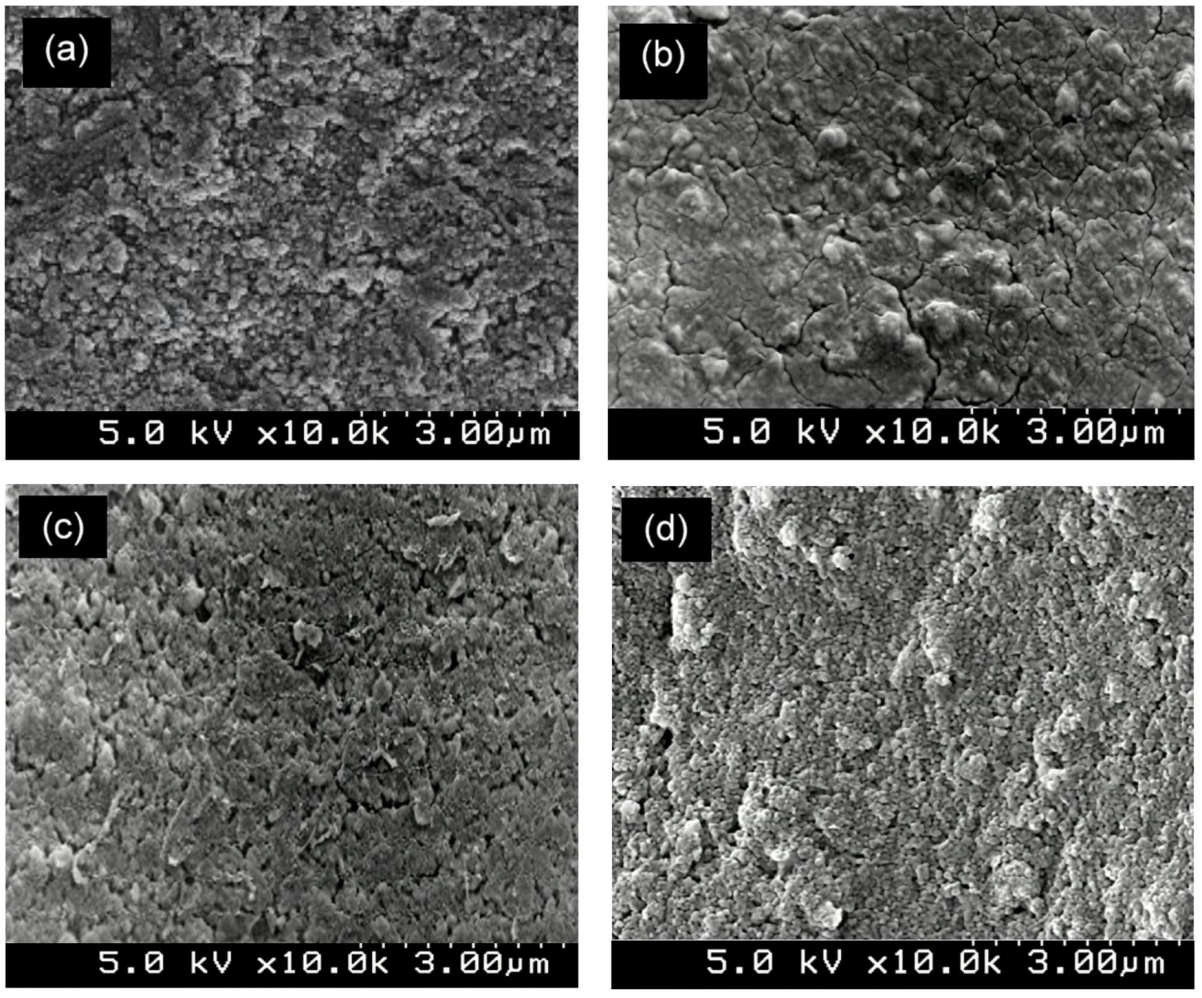
FESEM surface morphology of ZIF-67/AC/PDMS composite coatings with different filler concentrations: (**a**) 1 wt% (S2), (**b**) 3 wt% (S3), (**c**) 5 wt% (S4), and (**d**) 7 wt% (S5).

The surface morphology of the fabricated ZIF-67/AC/PDMS composite coatings was investigated using FESEM to understand the influence of filler concentration on the surface roughness and porosity. As illustrated in Fig. 3(a), at a low filler concentration (1 wt%, S2), the coating surface appears relatively smooth, with the hybrid particles partially embedded within the PDMS matrix. This indicates that the polymer binder predominantly covers the fillers, leading to limited surface roughness. With the increase of filler loading to 3 wt% (S3) and 5 wt% (S4), the surface topography transitions into a more irregular and fractured appearance, as shown in Fig. 3(b) and (c). The hybrid ZIF-67/AC particles begin to emerge through the polymer layer, creating micro-scale protrusions. The most significant morphological change is observed in the optimized sample (7 wt%, S5), shown in Fig. 3(d). The surface is characterized by a dense, hierarchical nano-granular structure, where the ZIF-67/AC hybrid fillers are uniformly distributed and effectively exposed. The exposed fillers create a “re-entrant geometry” that facilitates the entrapment of air pockets beneath the water droplets, effectively supporting the Cassie-Baxter mechanism.

### FTIR analysis

**Fig. 4:**
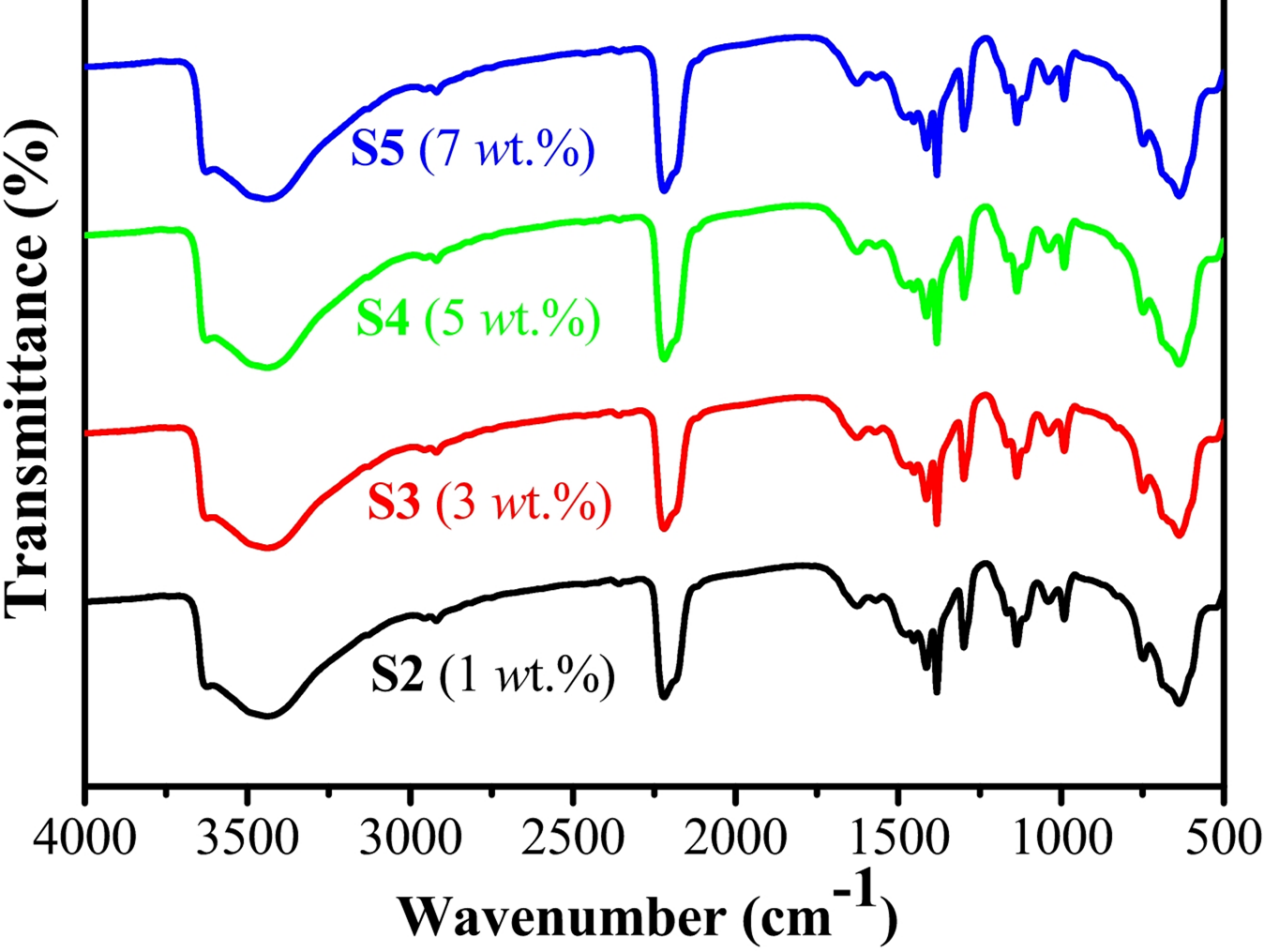
FTIR of ZIF-67/AC/PDMS composite coatings with different filler concentrations: S2 (1 wt%), S3 (3 wt%), S4 (5 wt%), and S5 (7 wt%).

Figure 4 (FTIR spectra) illustrates the chemical functional groups of the ZIF-67/AC/PDMS composite coatings at different filler concentrations (S2–S5). A broad absorption band observed at approximately 3440 cm⁻¹ is attributed to the O–H stretching vibrations, likely originating from adsorbed moisture or surface hydroxyl groups on the activated carbon. The sharp peak at 2230 cm⁻¹ is a characteristic feature often associated with the framework of the hybrid composite. The fingerprint region (1500–600 cm⁻¹) further confirms the successful integration of all components. Specifically, the characteristic vibrations of the imidazole ring from ZIF-67 are visible between 600 and 1500 cm⁻¹. The prominent band around 1010–1090 cm⁻¹ corresponds to the Si–O–Si stretching vibration, confirming the presence of the PDMS matrix. Furthermore, the peak at 1260 cm⁻¹ is assigned to the Si–CH₃ symmetric bending, while the absorption at approximately 425 cm⁻¹ (noted at the end of the spectra) confirms the Co–N stretching vibration, providing evidence for the structural integrity of the ZIF-67 fillers within the polymer matrix. The high similarity across all samples (S2–S5) indicates that the chemical composition remains consistent despite the increase in filler loading.

### Surface wettability and chemical durability analysis

**Fig. 5:**
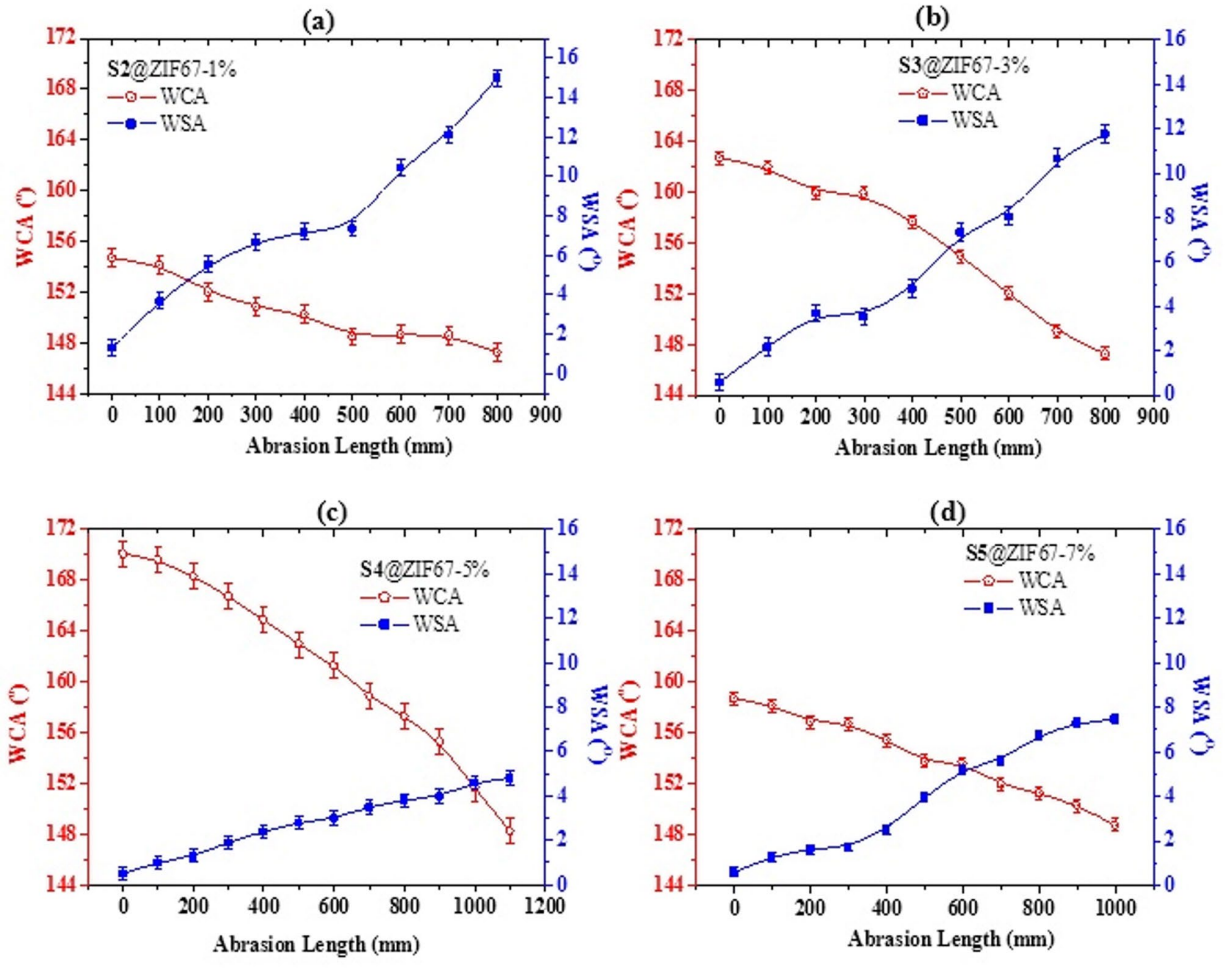
Variation of WCAs and WSAs as a function of abrasion length for the SHP-coated steel samples: (**a**) S2@ZIF67-1%, (**b**) S3@ZIF67-3%, (**c**) S4@ZIF67-5%, and (**d**) S5@ZIF67-7%.

The wetting properties of the fabricated coatings were evaluated by measuring the Water Contact Angle (WCA) and Sliding Angle (SA). The transition from a hydrophilic steel surface to a superhydrophobic state was systematically studied across the prepared samples S1 to S5. Effect of ZIF-67 concentration on WCA The relationship between ZIF-67 loading and the static WCA is illustrated in Fig. 5. Sample S1 (Bare Steel) exhibited a typical hydrophilic nature with a WCA of approximately 52^o^. Sample S1 (0% ZIF-67/AC/PDMS) introduction of AC and PDMS increased the WCA to 125^o^, demonstrating inherent hydrophobicity due to the low surface energy of PDMS and the nanoroughness of AC. Samples S2 - S3 (1% − 3% ZIF-67) a sharp increase in WCA was observed as the ZIF-67 concentration increased. The nanocrystals began to provide the necessary nanoscale roughness, trapping air pockets according to the Cassie-Baxter model^24^. The Optimized State S4 has maximum hydrophobic performance was achieved at 5 wt% ZIF-67, where the WCA reached an impressive 168^o^ with a sliding angle (SA) of less than 5^o^. At this concentration, the hierarchical nanostructure is perfectly balanced, creating a stable “plastron” air layer that prevents water droplets from penetrating the surface^25^. Overloading Effect (S5@7% ZIF-67). Interestingly, further increasing the ZIF-67 concentration to 7% (S5) resulted in a slight decline in WCA (down to 154^o^). This is attributed to the agglomeration of nanoparticles at high loadings, which disrupts the uniform hierarchical roughness and creates larger clusters that water droplets can partially “pin” to, transitioning slightly toward the Wenzel state^26,27^. While the 48-hour immersion period provides a snapshot of the coating’s initial chemical resilience, the sustained superhydrophobicity and stable contact angles suggest a robust resistance to wetting transitions. Future studies will focus on extended long-term exposure to further evaluate the life-cycle of these composite coatings under continuous industrial stress.

**Fig. 6:**
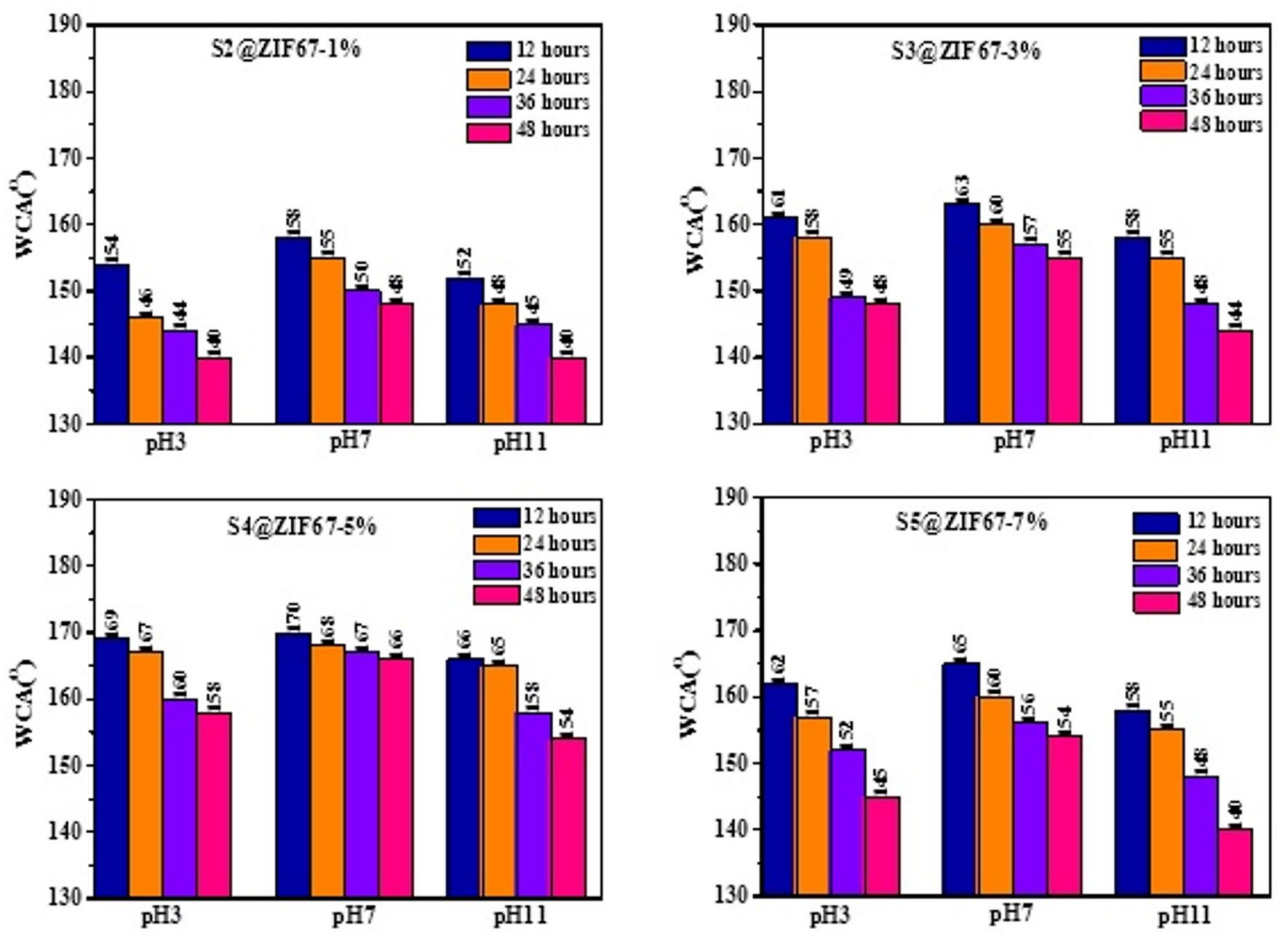
Comparison of chemical stability for samples S2, S3, S4, and S5 at different pH values (3, 7, and 11) and immersion times (12, 24, 36 and 48 h).

The chemical durability of the fabricated ZIF-67/AC/PDMS coatings with varying MOF concentrations (S2–S5) was systematically investigated across pH range 3, 7, and 11 over time domains 12, 24, 36 and 48 h, as illustrated in Fig. 6. The results showed a clear correlation between the ZIF-67 and the maintenance of superhydrophobic properties. For sample S2 (1 wt%), significant decrease in water contact angle (WCA) was observed. Notably that post 24 h, the recorded WCA values were below the superhydrophobic threshold 150^o^ in both acidic and alkaline media indicating insufficient ZIF-67 concentration needed for exact roughness maintaining stable Cassie-Baxter state under chemical stress^28^. Regarding 3 wt% (S3) and 7 wt% (S5) concentrations, better WCAs angles were obtained at 12 h. Moreover, S5 showed sharper decline in WCA when compared to S4, reaching 140° at pH 11 after 48 h. Such performance in S5 results could be due to the agglomeration of excess nanocrystals. This agglomeration result in nanocracks and defects in the PDMS matrix. In turn, enabled corrosive ions to penetrate within the coating presistently. In contrast, the optimized 5wt.% (S4) sample exhibited the most robust chemical resilience. It maintained its superhydrophobicity across all pH values throughout the 48 h test, with final WCAs of 158°, 166°, and 154° for pH 3, 7, and 11, respectively. The superior performance of S4 confirms that 5 wt% is the critical concentration that achieves the perfect balance between surface roughness and polymer encapsulation^29^. The PDMS binder in S4 effectively surrounds the well-dispersed ZIF-67/AC particles, creating a durable chemical shield that prevents the dissociative attack of $\:{H}^{+}\:$and $\:{OH}^{-}$ ions on the structural framework. The sustained superhydrophobicity after immersion in various pH solutions (3–11) indicates that the ZIF-67/AC framework remains structurally intact, as any chemical degradation would have compromised the surface micro/nano-topography required for the Cassie-Baxter state.

### Mechanical durability analysis

**Fig. 7:**
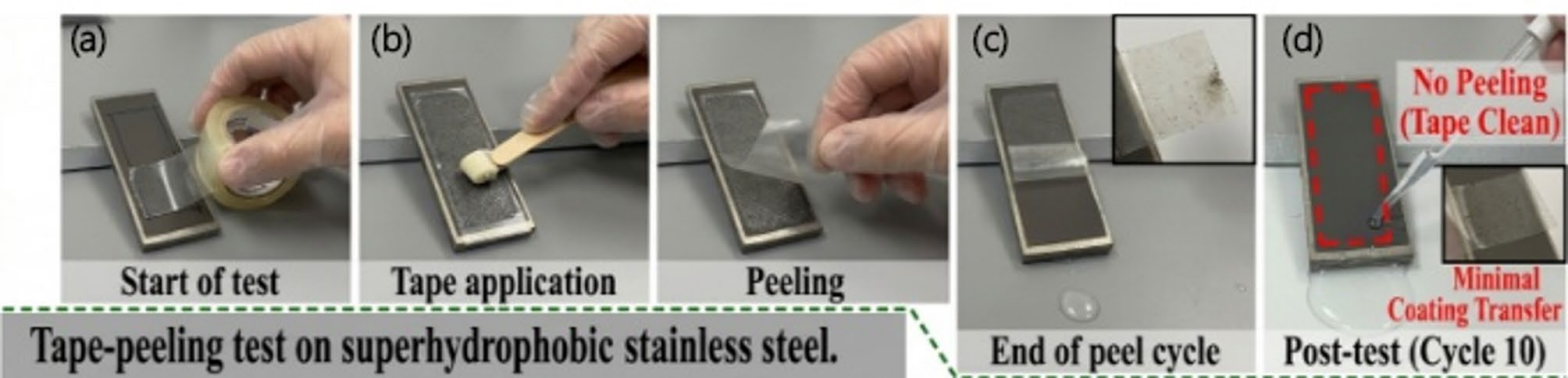
Mechanical durability assessment for the ZIF-67/AC/PDMS hybrid coating (S5) using the tape-peeling test (ASTM D3359). The panels illustrate (**a**) the start of the test on the coated steel, (**b**) the application of adhesive tape with uniform pressure using a roller, (**c**) the peeling process, and (**d**) the surface appearance after 10 peeling cycles.

To evaluate the long-term mechanical stability and adhesion of the ZIF-67/AC/PDMS hybrid coating on the steel substrate, a tape-peeling test was conducted (Fig. 7). The process involved applying an adhesive tape under uniform pressure, followed by its rapid removal. Despite the high adhesive force of the tape, the coating remained intact with minimal coating transfer, as evidenced by the clean tape appearance after 10 cycles. The maintenance of the ‘Cassie-Baxter’ state post-testing was confirmed by the water droplet bouncing behavior indicates that the hierarchical micro-nano structure is securely anchored. This robustness is attributed to the PDMS binder, which acts as a flexible yet strong bridge, providing mechanical interlocking between the porous hybrid fillers and the roughened steel surface.

### Corrosion resistance behavior

**Fig. 8:**
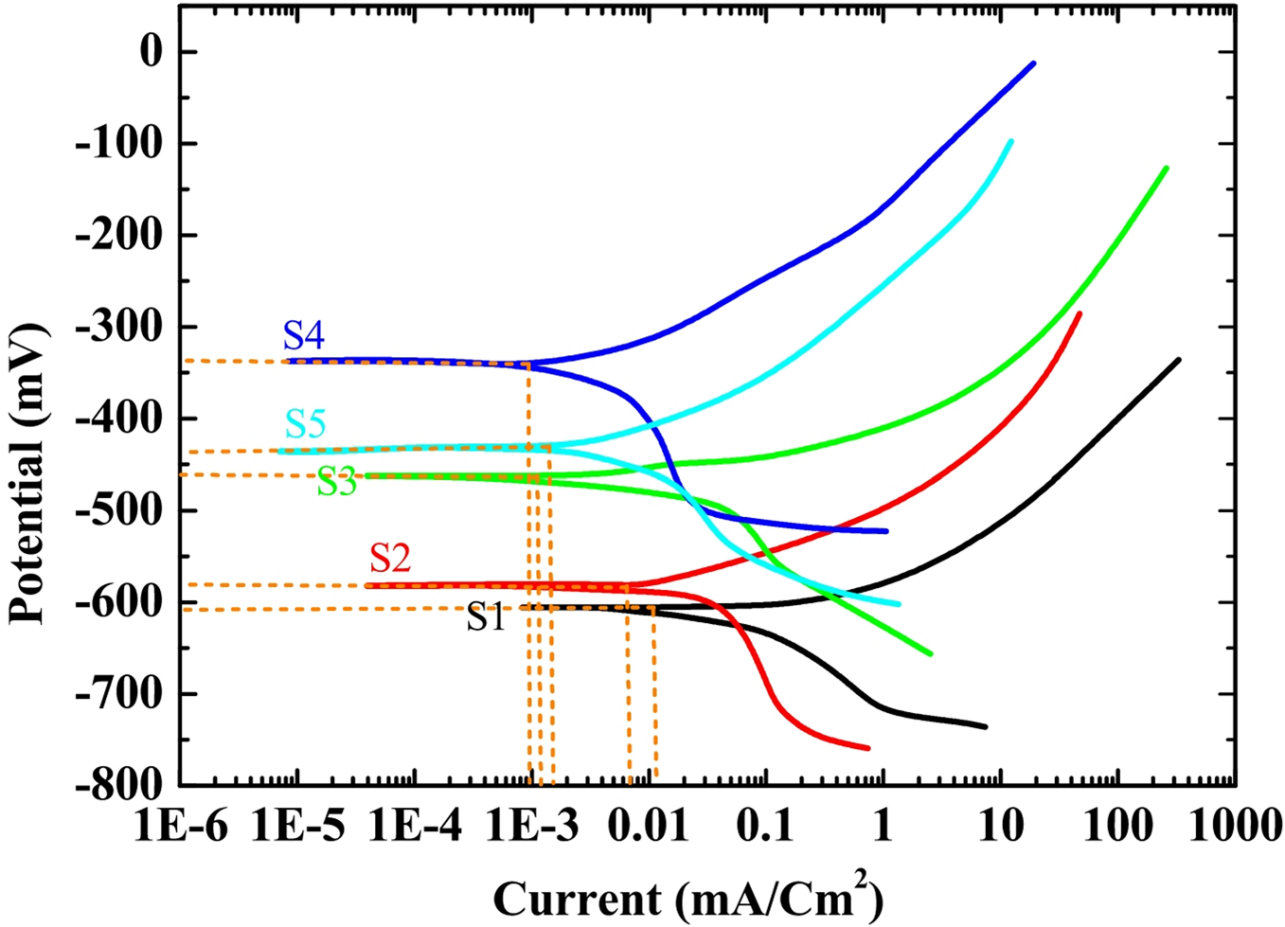
Potentiodynamic polarization (PDP) curves of bare steel and ZIF-67/AC/PDMS nanocomposite coatings with different loadings.

The corrosion mitigation performance of the developed ZIF-67/AC/PDMS coatings was quantitatively assessed using potentiodynamic polarization (PDP) in a marine-simulating environment (3.5 *wt*% NaCl) Potentiodynamic Polarization (PDP) Analysis. The polarization curves (Tafel plots) for the bare steel and the various SHP-coated samples (S1–S5) are illustrated in Fig. 8. The cathodic branch of the curves represents the oxygen reduction reaction, which is the primary cathodic process in aerated neutral saline solutions, as shown in Eq. (2)^30^.

2 $$\:{O}_{2}\:+\:2{H}_{2}O\:+\:4{e}^{-}\to\:\:4O{H}^{-}$$

The electrochemical parameters derived from the Tafel extrapolation method, including corrosion potential E_corr_, anodic/cathodic Tafel slopes β_a_, β_c_ and corrosion current density i_corr_, are summarized in Table 3. The protection efficiency %P was calculated based on the i_corr_ values using Eq. (3)^31^.

3 $$\:\%P=\left(1-\frac{{i}_{corr}}{{i}_{corr}}\right)*100$$

Where i_corr_ and i_corr_ are the current densities of the bare and coated steel, respectively. The mechanical robustness of the fabricated coatings was comparatively investigated across various ZIF-67 loadings (S2-S5). As illustrated in the comprehensive abrasion profiles, the S2 (1%) sample displayed the poorest durability, with a rapid decline in superhydrophobicity and an early intersection point at approximately 200 mm. In contrast, increasing the nanocrystal loading to 5% (S4) significantly enhanced the structural integrity, delaying the failure threshold to over 1000 mm. This remarkable durability is attributed to the optimized interlocking between the ZIF-67 rhombic dodecahedra and the PDMS matrix^32^, providing a resilient hierarchical shield against mechanical shear. Beyond this concentration (S5, 7%), a decline in performance was observed, likely due to particle agglomeration which creates mechanical weak points in the coating. Figure 8 illustrates the Potentiodynamic Polarization (PDP) curves for bare steel and other fabricated coatings immersed in a 3.5 wt% NaCl solution. It is evident that all coated samples exhibited significant shift towards more noble potentials E_corr_ and a dramatic reduction in corrosion current densities I_corr_ compared to the bare steel substrate^33^. Among the tested formulations, the optimized S4 sample 5 wt% ZIF-67 demonstrated the most superior corrosion resistant sample. The E_corr_ was found to shift positively to approximately 340 mV, while the I_corr_ was observed to drop by several orders of magnitude, reaching a minimum of $\:0.05\times\:$ 10^− 3^ µA/cm^2^. This remarkable performance is attributed to the synergistic effect of the superhydrophobic ZIF-67/AC and the insulating PDMS binder, which effectively creates a robust barrier preventing the infiltration of corrosive $\:{Cl}^{-}$ ions to the steel surface^34^. On the other hand, the performance of the other samples showed a slight decline, likely due to the over-saturation of nanocrystals leading to minor surface defects, which is consistent with the mechanical durability results. The anodic β_a_​ (mV/decade) and cathodic β_c_​ (mV/decade) Tafel slopes were extracted from the linear portions of the polarization curves, see Table 3. The variation in these slopes upon coating application indicates that the ZIF-67/AC/PDMS film acts as a mixed type inhibitor, retarding both metal dissolution and cathodic reduction reactions. The stability of the Cassie–Baxter state during immersion is further supported by the EIS data. The maintenance of high impedance values indicates that the air plastron layer effectively remains trapped within the hierarchical ZIF-67/AC/PDMS structure, acting as a physical barrier that prevents the NaCl electrolyte from reaching the metal substrate. This is also consistent with the optical ‘silver mirror’ effect observed on the coating surface during immersion, which is a clear indicator of a persistent air-liquid interface.

**Table 3:** Electrochemical corrosion parameters extracted from the PDP curves for bare and SHP-coated steel in 3.5 wt% NaCl solution.

Sample	−E_corr_​ (mV)	β_a_​ (mV/decade)	−β_c_​ (mV/decade)	i_corr_​ (µA/cm^2^)	%*P* (Efficiency)
Bare steel	610.15	95.4	120.3	32.5	–
S1	585.42	110.25	135.6	6.45	80.15
S2	560.1	125.1	148.2	2.1	93.53
S3	465.35	140.55	165.8	1.05	96.76
S4	340.22	185.3	210.45	0.05	99.84
S5	435.18	138.4	170.12	1.15	96.46

### Electrochemical impedance spectroscopy (EIS) and equivalent circuit modeling

**Fig. 9:**
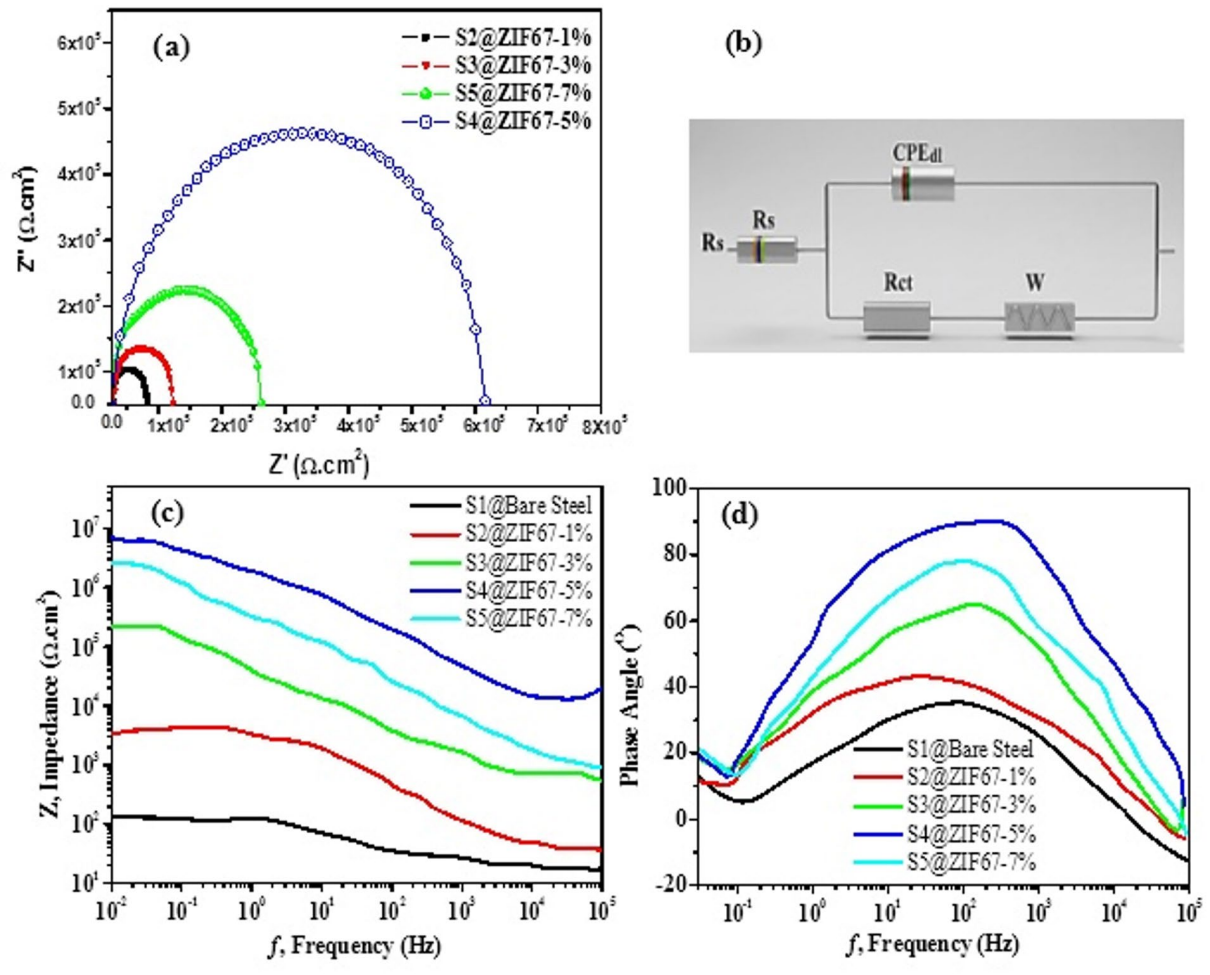
Electrochemical Impedance Spectroscopy (EIS) analysis for bare steel and ZIF-67/AC/PDMS coated samples in 3.5 wt% NaCl solution (**a**) Nyquist plots, (**b**) the corresponding equivalent electrical circuit (EEC) used for data fitting, (**c**) Bode impedance plots |Z| vs. frequency, and (**d**) Bode phase angle plots θ vs. frequency for samples S1–S5.

To further investigate the corrosion protection mechanism, the EIS data were fitted using a two time constant equivalent electrical circuit (EEC), as illustrated in Fig. 9. The 3D model depicts the hierarchical structure of the ZIF-67/AC/PDMS composite. In this model, R_s_ represents the solution resistance. The first time constant R_c_/CPE_c_ is associated with the dielectric properties and pore resistance of the superhydrophobic layer^35^. The second time constant R_ct_/CPE_dl_ characterizes the electrochemical reactions at the steel/electrolyte interface. The constant phase element (CPE) was utilized to account for the surface nonhomogeneity and fractal roughness induced by the ZIF-67 nanocrystals and activated carbon particles. The high frequency time constant represents the barrier properties of the hybrid coating, while the low frequency response characterizes the charge transfer process at the metal surface.

The Bode impedance plots Fig. 9(a) provide further insight into the protective properties of the coatings. At low frequencies 10^− 2^ Hz, the impedance modulus |Z| of the optimized sample S_4_ reached its maximum value of approximately$\:\:8\times\:{10}^{6}\:$Ω.cm^2^, which is several orders of magnitude higher than that of the bare steel and other samples. This significant increase in impedance confirms the superior barrier performance and the robust superhydrophobic nature of the ZIF-67/AC/PDMS composite^36^. Crucially, a direct correlation exists between the maximum WCA (162^o^) and the |Z|_0.01 Hz_ values, as the trapped air plastron significantly increases the total resistance of the system. The experimental data showed an excellent fit with the proposed equivalent circuit model, where the presence of the Warburg element (W) suggests a diffusion controlled corrosion mechanism through the hierarchical porous structure of the coating.

Furthermore, the coating porosity (P) and water uptake were calculated based on the capacitance data to support the Bode findings. Sample S4 exhibited the lowest water uptake (less than 1.2%), which correlates perfectly with the high-frequency plateau observed in the Bode modulus plots, indicating a dense, nearly impermeable barrier.

**Table 4:** Electrochemical parameters obtained from fitting the EIS experimental data using the (R_s_ Q_dl_)/(R_ct_ .W) equivalent circuit model.

Sample	*R*_s_​ (Ω⋅ cm^2^)	CPE_dl_​×10^− 6^ (S⋅ s^*n*^⋅ cm^− 2^)	*N*	*R*_ct_​ (Ω⋅ cm^2^)	W×10^− 6^ (S⋅ s^0.5^⋅ cm^− 2^)
S2	25 ± 2	8.5	0.82	7.2 × 10^4^	45.2
S3	28 ± 3	5.2	0.85	1.1 × 10^5^	32.1
S5	30 ± 2	3.1	0.88	2.5 × 10^5^	15.4
S4	32 ± 4	0.9	0.94	6.1 × 10^5^	5.8

The electrochemical parameters derived from fitting the experimental EIS data using the R_s_ Q_dl_ /R_ct_ W equivalent circuit model are summarized in Table 4. As shown in the table, the numerical values for the charge transfer resistance R_ct_ further support the visual observations in the Bode plots Fig. 9(c), where sample S4 demonstrated a two-order of magnitude improvement in corrosion protection compared to the base coating. This dramatic rise in R_ct_ for sample S4 compared to S2 and S3 highlights the synergistic effect of the ZIF-67 nanocrystals and activated carbon (AC) in enhancing the all corrosion resistance of the PDMS matrix^37^.

The decrease in CPE_dl_ and increase in Rct serve as a quantitative confirmation that the ‘Air Plastron prevents the electrolyte from reaching the steel, effectively reducing the electrochemically active surface area.

Furthermore, the notable decrease in the constant phase element CPE_dl_ values for the optimized coatings suggests a significant reduction in the electrochemically active surface area. This effect is primarily attributed to the robust superhydrophobic protection, which effectively minimizes the contact area between the corrosive electrolyte and the steel substrate. Consequently, the combination of high R_ct_ and low CPE_dl_ confirms that the S4 composite coating acts as an exceptional barrier against ion penetration, providing robust chemical resistance durability in harsh environments. The phase angle evolution as a function of frequency, Fig. 9(d), provides critical information regarding the capacitive nature and structural integrity of the prepared coatings. As illustrated in the Bode phase plots, the optimized sample S4 (ZIF-67/AC/PDMS) exhibits a remarkably broad and high phase angle peak, approaching 90^o^ in the intermediate frequency range 10 to 10^3^ Hz. This ideal capacitive behavior is a direct indicator of a purely capacitive response^38,39^, confirming that the coating acts as an impenetrable physical barrier against the electrolyte. The persistence of this high phase angle over a wide frequency spectrum for sample S4 compared to the significantly lower and narrower peaks of the bare steel and other composite ratios signifies the formation of a dense, defect-free, and superhydrophobic protective layer. This response suggests that the synergistic combination of ZIF-67 nanocrystals and activated carbon within the PDMS matrix effectively seals the micropores, preventing the diffusion of corrosive species such as $\:{Cl}^{-}$ and O_2_towards the metal surface. Consequently, the phase angle data reinforces the Nyquist and |Z| results, highlighting S4 as the most robust configuration for corrosion protection.When compared to recent literature (2021–2026) on superhydrophobic coatings, the developed ZIF-67/AC/PDMS system shows a significant improvement. While recent studies using ZIF-8 or GO-based coatings reported WCAs in the range of 152°–158°, our hybrid composite achieved 162°. Furthermore, the electrochemical stability and Rct values recorded in this work are among the highest reported for MOF-polymer hybrids on steel substrates, confirming the effectiveness of the dual-filler strategy.

### Evaluation of anti-scaling performance and mineral resistance

The integration of anti-scaling properties is crucial for maritime and industrial applications, as mineral deposits like CaCO_3_ often trigger under deposit corrosion. In this study, the anti-scaling evaluation was conducted in a supersaturated solution (0.5 M CaCl_2_ + 0.5 M NaHCO_3_) at an accelerated temperature of 50^o^ C. The results confirm that the ZIF-67/AC/PDMS coating acts as a dual-action barrier; the hierarchical roughness not only prevents corrosive ion diffusion but also drastically reduces the nucleation energy required for scale formation.

**Fig. 10:**
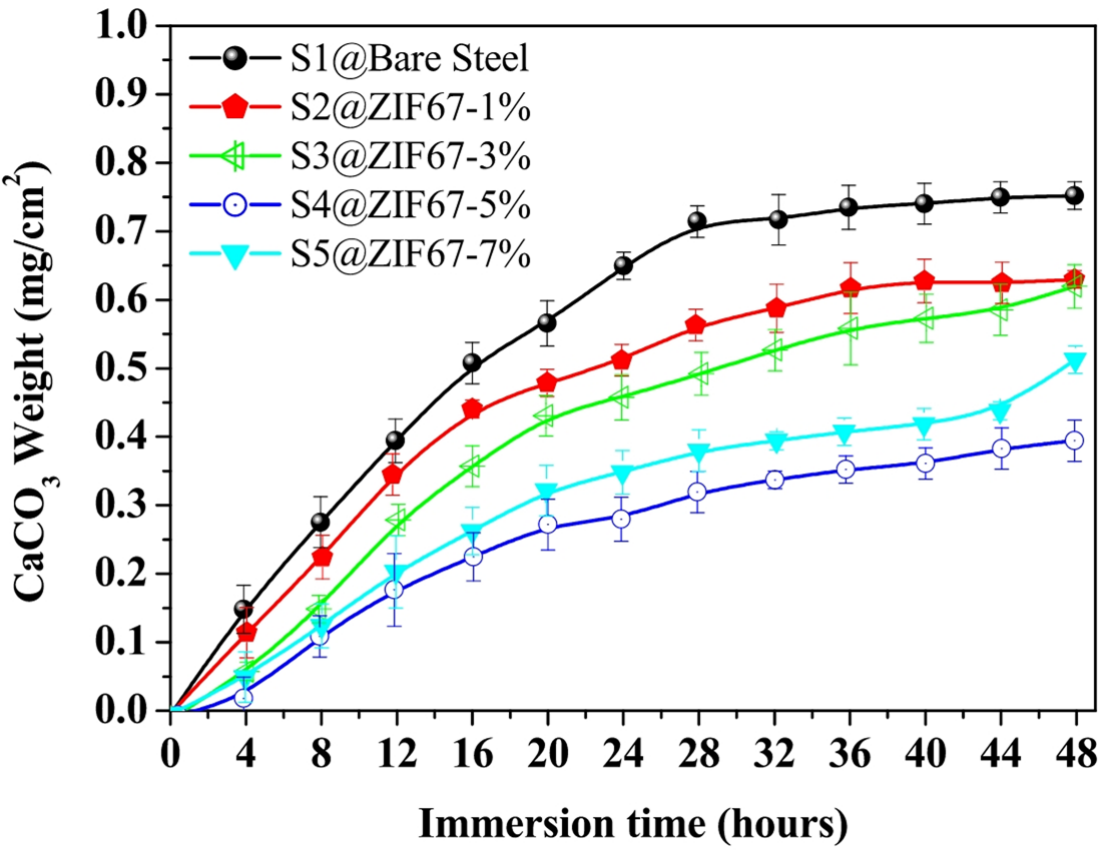
Time dependent accumulation of CaCO_3_ deposits on all samples S1, S2, S3, S4 and S5 ZIF-67/AC/PDMS composite coatings during a 48 h immersion period.

The antiscaling performance was quantitatively evaluated by measuring the mass of CaCO_3_ per unit area mg/cm^2^. Sample S4 (5 wt% ZIF-67) exhibited the most significant scaling inhibition, maintaining a remarkably low deposit weight of approximately 0.39 mg/cm^2^ at the 48 h mark, representing a nearly 50% reduction compared to bare steel. The enhanced resistance to mineral adhesion is attributed to the synergistic effect of the superhydrophobic surface and the air trapping Cassie Baxter state, which minimizes the nucleation sites for crystal growth. Error bars represent the standard deviation from three independent measurements, confirming the reproducibility of the anti-scaling results. The antiscaling evaluation, Fig. 10, aligns with the electrochemical and wettability stability results. The persistent superhydrophobicity observed in the 48 h WCA tests directly correlates with the lower scaling rates. The optimized composite structure effectively resists the wetting transition, ensuring that the trapped air layer remains as a barrier not only against corrosive ions but also against the nucleation and adhesion of mineral scales like CaCO_3_.

### Proposed anti-corrosion and anti-scaling mechanism

**Fig. 11:**
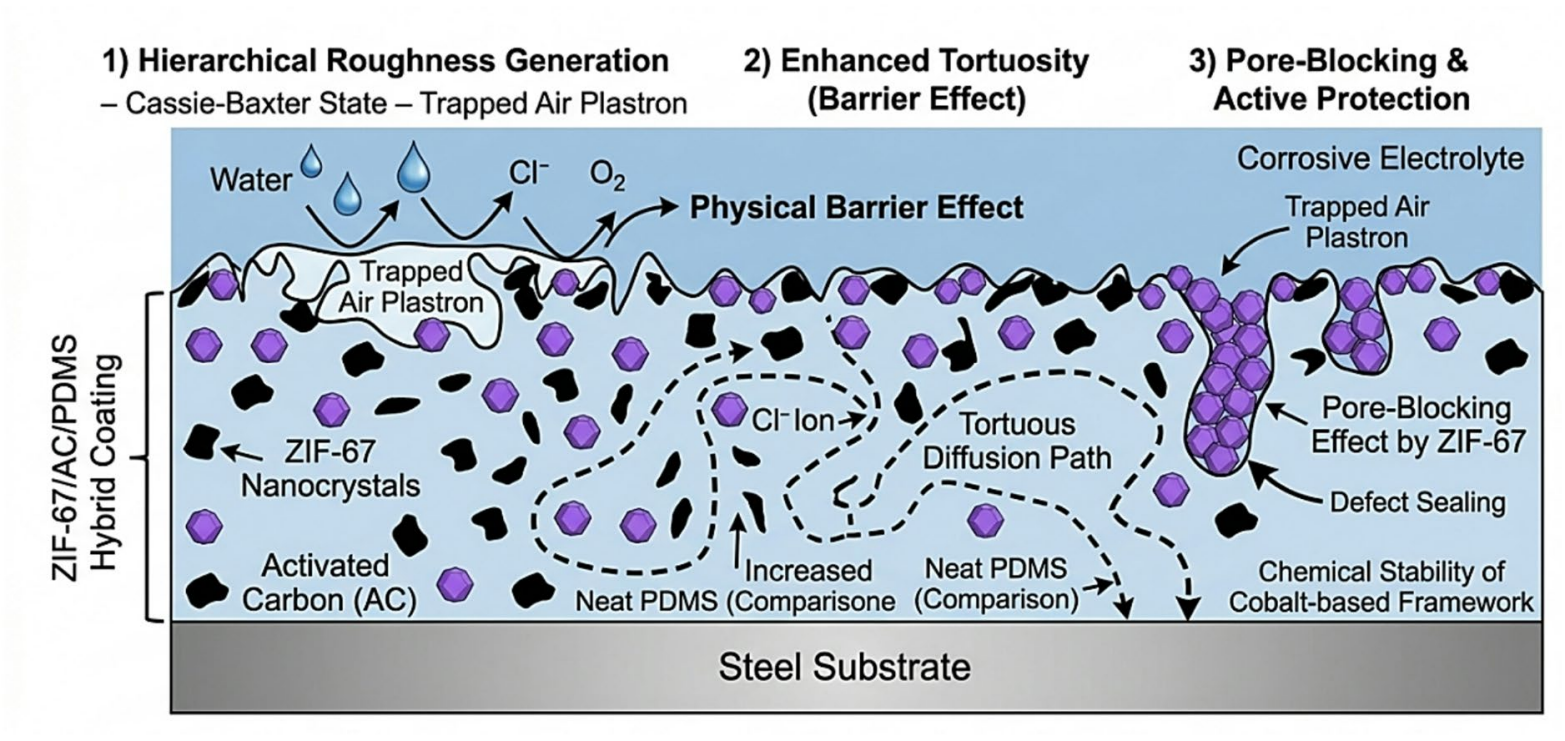
Schematic illustration of the triple-action synergistic mechanism (Hierarchical roughness, Tortuous barrier, and Pore-Blocking) for the ZIF-67/AC/PDMS hybrid coating on steel.

Based on the cumulative findings from morphological, electrochemical, and anti-scaling assessments, a comprehensive mechanism for the ZIF-67/AC/PDMS hybrid coating is proposed, as illustrated in Fig. 11. The synergistic integration of ZIF-67 nanocrystals and AC fillers within the PDMS matrix creates a robust hierarchical roughness that stabilizes the Cassie-Baxter air plastron. This performance stems from a direct correlation between the structural integrity of the ZIF-67 framework (verified by XRD) and the micro-scale surface topography (confirmed by FESEM), which together sustain the superhydrophobic state. This trapped air layer acts as a primary physical barrier that not only repels corrosive species like $\:{Cl}^{-}$ and $\:{O}_{2}\:$through an enhanced tortuous path but also minimizes the nucleation sites available for CaCO_3_ crystal growth. Furthermore, the pore blocking effect provided by the ZIF-67 framework seals micro defects in the polymer binder, ensuring long-term chemical resilience and structural integrity in harsh environments. The high charge transfer resistance (Rct) observed in EIS measurements further validates this synergistic mechanism, highlighting the effective barrier properties afforded by the hybrid composite.

## Conclusion

A high performance superhydrophobic coating based on a ZIF-67/AC/PDMS nanocomposite was successfully fabricated on steel substrates using a controllable dip-coating technique. The investigation of varying MOF concentrations revealed that the S4@5wt.% ZIF-67 formulation represents the critical threshold for achieving a perfect balance between surface roughness and structural integrity. Morphological analysis via TEM and PSA confirmed the synthesis of exact ZIF-67 rhombic dodecahedra with an optimized average particle size of 30 nm, which played a pivotal role in stabilizing the Cassie-Baxter state. Consequently, sample S4 exhibited an extraordinary water contact angle of 170° and demonstrated remarkable chemical resilience across a wide pH3 and pH11 for up to 48 h. Electrochemical evaluations PDP and EIS further validated the superior protective nature of the S4 coating, which yielded a protection efficiency of 99.84% and a high charge transfer resistance R_ct_, effectively shielding the steel from corrosive ions in a simulated marine environment. Additionally, the coating displayed robust mechanical durability, withstanding over 1000 mm of abrasion, and significant anti-scaling properties with a 50% reduction in CaCO_3_ accumulation compared to bare steel. These results demonstrate that the S4 composite coating is a highly promising candidate for industrial applications requiring robust chemical resistance anti-corrosion and anti-scaling protection in harsh environments.

## Data Availability

The data that support the findings of this study are available from the correspondingauthor upon reasonable request.
